# Beliefs, Practices, and Knowledge of Household Food Handlers Regarding the Impact of Electricity Outages on Food Safety: Findings from a National Cross-Sectional Study in Lebanon

**DOI:** 10.3390/foods14050855

**Published:** 2025-03-02

**Authors:** Noura Subuh, Rouba Ballout, Imad Toufeili, Issmat I. Kassem, Samer A. Kharroubi

**Affiliations:** 1 Department of Nutrition and Food Sciences, Faculty of Agricultural and Food Sciences, American University of Beirut, P.O. Box 11-0236, Beirut 1107-2020, Lebanon; noa13@mail.aub.edu (N.S.); rb166@aub.edu.lb (R.B.); toufeili@aub.edu.lb (I.T.); issmat.kassem@uga.edu (I.I.K.); 2 Center for Food Safety, College of Agricultural and Environmental Sciences, University of Georgia, Athens, GA 30602, USA

**Keywords:** food safety, foodborne illness, electricity outage, food handler, knowledge, beliefs, practices, Lebanon

## Abstract

Food safety continues to be a global concern threatening human life, especially in low-income countries where frequent electricity outages pose higher risks to food safety, increasing the risks of foodborne illnesses due to temperature fluctuations. This study aimed to assess the consumer’s knowledge of food safety, beliefs, and household practices during electricity cut-off. A cross-sectional study among consumers in Lebanon was conducted using an online survey (n = 571). Results revealed that food handlers in Lebanon had unsatisfactory food safety knowledge levels along with poor food safety beliefs and practices. The findings also showed that good knowledge scores were significantly associated with age, governorate, educational level, a self-reported food safety knowledge score, and the frequency of checking the temperature of fridges/freezers (*p* < 0.001). This study exposed inadequate food safety knowledge and deficient food safety-related beliefs and practices among participants in Lebanon, particularly during periods of electricity outages. These gaps highlight the need for educational interventions and structured efforts to enhance participants’ understanding of safe food handling and storage practices under challenging conditions to reduce the risk of foodborne illnesses and improve the public health outcomes in Lebanon.

## 1. Introduction

Despite numerous scientific and technological efforts, food and waterborne illnesses continue to threaten humans and the economy. Though global, this issue is particularly serious in low-income countries [1]. Many challenges compromise the global system in providing safe food and water. These challenges include environmental changes (climate and water scarcity), population growth, food production changes, the emergence of new pathogens, and contaminants, especially antibiotic resistance and economic crises [2,3]. Despite the increase in knowledge of food safety, there is still a major gap due to the insufficiency of awareness among populations regarding the safety of food, especially since food can be contaminated at any stage of the food chain from receiving to consumption and can result in the serious risk of foodborne diseases [2].

Foodborne acute gastroenteritis is often associated with improper food handling practices in households. These include infrequent or incomplete heating, inadequate food storage, cross-contamination, and the presence of infected food handlers [4]. Moreover, substandard food safety knowledge and practices among home food handlers can lead to food contamination, which is a significant cause of foodborne illness [5]. This can be compounded by frequent and long electricity outages that render the storage of high-risk food particularly troublesome since bacteria can multiply rapidly when these foods are left within the temperature danger zone (5–60 °C) with bacterial counts reaching dangerous levels if kept for more than 2 h [6]. For instance, Langiano et al., (2012) noted that the inadequate understanding of foodborne illnesses and pathogens persists among families in Italy with poor hygienic practices was also observed during the preparation and storage of food [7]. A study conducted in South Africa examined the food safety practices of household food handlers, who play a vital role in maintaining household food safety. The findings revealed that, while handwashing and surface cleaning practices were generally adequate, food storage practices—especially for meat, chicken, and fish—were insufficient [8].

Lebanon is in a critical situation due to the many crises that this country has been through. Starting with the civil war to the current economic meltdown, political instability, the disproportionately large number of refugees, and the COVID–19 pandemic wreaked havoc on the food sector [9]. Moreover, the absence of a public water supply in Lebanon might drive households to compromise their basic water, sanitation, and hygiene requirements, which can affect their food handling practices with a concomitant increase in foodborne diseases [10]. In Lebanon, food and water-related outbreaks are usually detected when “spatiotemporal clusters” are confirmed, or the history of exposure reveals commonly consumed meals. Thus, distributed outbreaks are not accounted for and are mostly hidden by the endemicity of the illness [11]. In some countries, homes account for the highest percentage of foodborne illness outbreaks. The analysis of data on foodborne outbreaks in Brazil (2000–2018) identified households as the most frequent location of foodborne illnesses (45.6%) [12]. In the European Union, 36.4% of reported outbreaks were traced back to households [13].

A study in Lebanon indicated that there is a lack of food safety awareness among young participants regarding several practices and attitudes including cross-contamination, cooking, thawing, and prevention procedures [14]. According to Hassan et al., only 35.8% of Lebanese food handlers knew that freezing food cannot kill bacteria, and 54.5% agreed on placing the prepared food in the fridge and reheating the food if not consumed within three hours [15]. In times of emergency, like electricity cut-off, not only are the quality and safety of food affected but also the dietary habits and food choices [16]. Another critical issue in Lebanon is water contamination and its accompanying effects on irrigation quality and agricultural products [17]. Many of Lebanon’s rivers, including the main Litani River, and groundwater are polluted with untreated sewage and leaks from unregulated dumpsites [18]. Notably, a study in Lebanon detected 22 mcr-1 positive *E. coli* in irrigation water samples from major agricultural regions in the country with the isolates being resistant to penicillin, ampicillin, and tetracycline [19]. This strongly suggests that ready-to-eat foods like fruits and vegetables are a source of contamination and potentially causative agents of foodborne illness.

Nine agencies govern food safety in Lebanon. The lack of coordination among these agencies coupled with the absence of accountability is believed to have had grave consequences that impacted the safety and quality of food products in the country. These agencies operate under vague food safety laws, do not conduct regular food inspections, and often fall short of the effective control of microbiological and chemical hazards. The lack of awareness of many food businesses in Lebanon further jeopardizes public health and aggravates the poor food safety situation in the country [20,21].

The data on food safety knowledge among consumers during power outages are scarce. To date, one study has been conducted in the United States to assess the preparedness and understanding of the populations toward food safety during emergencies including electricity outages. The study found that only 15% of the participants were both prepared and knowledgeable about how to keep food safe during such situations [22]. Further, the study focused on emergencies, in general, and did not specifically address the frequent, and often daily, power outages in Lebanon recently, which calls for serious attention especially with the recent increase in food poisoning cases during the electricity cut-offs [23]. Therefore, the present study aims to assess the beliefs, practices, and knowledge regarding the impact of electricity outages on food safety among household food handlers in Lebanon. This study is significant as it is the first to investigate the impact of electricity outages on food safety in Lebanon. Electricity outages can disrupt food storage, refrigeration, and preparation, increasing the risk of foodborne illnesses. Despite the critical importance of this issue, research on its specific effects in Lebanon has been limited. By exploring how electricity outages affect food safety practices, this study fills a critical gap in the literature. Additionally, we examined the socio-demographic determinants of knowledge, beliefs, and practices related to this issue. Understanding these determinants is essential for developing targeted interventions and educational initiatives. By identifying key factors, this study aims to raise awareness, improve food safety practices, and reduce the risk of foodborne illnesses. Ultimately, our findings contribute to safeguarding public health by providing evidence-based insights to inform policy and strengthen food safety strategies in Lebanon.

## 2. Materials and Methods

### 2.1. Study Design and Sampling

A descriptive cross-sectional study was conducted between 17 February and 29 April 2022 among residents in Lebanon who are at least 18 years old. Sample size calculation was performed using the World Health Organization (WHO) sample size calculator that showed a minimum of 384 respondents who ought to be recruited to estimate a prevalence of 50% with a 95% CI and a margin of error of 5% [24]. To account for a 20% refusal rate, 576 respondents were then selected for this study.

### 2.2. Schematic Overview of the Survey Program

**Figure 1:**
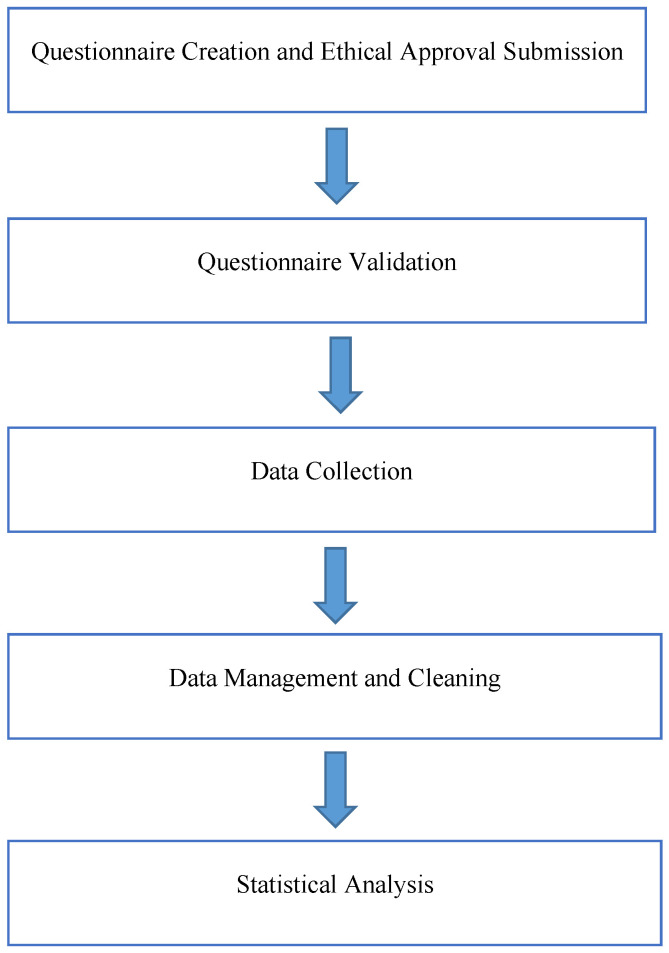
Schematic overview of the survey program.

Figure 1 presents a flow diagram outlining the key stages of the survey process, which comprised five primary steps: (1) developing the questionnaire and obtaining ethical approval, (2) validating the instrument, (3) recruiting participants and collecting data, (4) managing and cleaning the data, and (5) conducting statistical analysis. Each step was carefully designed to align with this study’s objective of assessing participants’ knowledge, attitudes, and practices regarding food safety during power outages. Moreover, these measures were implemented to minimize potential biases and enhance this study’s generalizability across different settings.

### 2.3. Survey Process

Due to social restrictions caused by the COVID–19 pandemic situation, this study was conducted online. The questionnaire was based on similar previous studies [15,22,25]. However, modifications were applied to assess the population’s knowledge and practices during electricity cut-off such as using recommendations published by the United States Department of Agriculture, Centers for Disease Control and Prevention, and Food Safety Government in the United States [26,27,28].

The survey was divided into 5 sections. The first section included questions related to the participant’s socio-demographic characteristics such as age, gender, area of residency, educational level, and total income. The second section was composed of basic questions related to food safety in households. The third section was related to knowledge about food safety. The fourth section was related to the attitudes towards the risks associated with food safety. The last section included questions about practices that could increase the risk of food poisoning.

### 2.4. Survey Validation

The questionnaire was validated using a two-step procedure. The instrument was first examined for content validity by a group of food science and food safety experts, who made sure that every item was understandable, pertinent, and in line with this study’s objectives. Second, a pre-testing was conducted where the survey was sent to 15 individuals to validate the questionnaire and to uncover any possible problems within the survey. However, those responses were not included in the analysis. The questionnaire’s generalizability was further improved by its design, which allowed it to be modified for comparable studies conducted in different locations.

### 2.5. Ethical Considerations

This study was conducted in accordance with the Declaration of Helsinki and approved by the Institutional Review Board (IRB) of the American University of Beirut (protocol code SBS-2022-0028 and date of approval 16 February 2022), and the research team was CITI-certified (Collaborative Institutional Training Initiative). The participants’ identities were completely anonymous; no name or any other personal information was recorded.

### 2.6. Data Collection

An online invitation (Supplementary Material File S1) was sent out via social media (WhatsApp groups, Facebook pages, Instagram), and participants were invited to this research. Before starting the questionnaire, a consent form (Supplementary Material File S2) appeared on their screen, which they could read and download. After that, once agreed to take part in this study, they proceeded to fill out the survey (Supplementary Material File S3). The completion of the questionnaire took approximately 10 min.

### 2.7. Statistical Analysis

The results obtained were statistically analyzed using the Statistical Package for the Social Sciences version (SPSS) 26.0. Complete responses were only used for the analysis. For the summary of the data, descriptive statistics were used. Frequencies and proportions were used for categorical variables. Means and standard deviations were used for continuous variables. Each multiple-choice question was given one point for the correct answer and zero points for the wrong answer and “I don’t know” answer. Total knowledge, beliefs, and practice scores were calculated for each participant by adding the number of correct answers. This results in a knowledge score ranging from 0 to 15, a practice score ranging from 0 to 20, and a belief score ranging from 0 to 6. The scores were then dichotomized. Participants with knowledge, beliefs, and practice scores below 70% were considered to have low levels, whereas those with scores ≥ 70% were considered to have high levels [29]. Chi-square test of independence was used to calculate the association between two categorical variables. The assumptions required for the chi-square test of independence, including expected frequency conditions, were assessed to ensure the validity of the analysis (Supplementary Material File S4). Regression analysis was performed to determine the predictor variables for consumer’s beliefs, practices, and knowledge levels. The socio-demographic characteristics represented the independent variables, whereas total knowledge, belief, and practice score ratings represented the dependent variables. Characteristics that showed statistical significance in the simple analysis were included in the multiple regression models as independent variables. A *p*-value (*p*) of 0.05 was considered.

## 3. Results

### 3.1. Participants Socio-Demographic Characteristics

A total of n = 571 participants completed the survey. The demographic characteristics of the respondents are summarized in Table 1. The sample was composed of 60.9% females (348) and 39.1% males (223). Participants’ ages ranged from 18 to 60 years and above, with the largest proportion (52.5%) belonging to the youngest age group (18–29). In terms of marital status, 57.4% of participants were single, 38.9% were married, and 3.8% were divorced, widowed, or separated. Nearly half of the respondents (49%) resided in Mount Lebanon, while 28.9% lived in Beirut, followed by 9.8% in the South, 8.4% in the North, and 4.0% in the Bekaa region. Regarding educational attainment, 70.8% of the participants held a university degree, including bachelor’s, master’s, or PhD qualifications. For household income, 57.8% reported a monthly income below LBP 10,000,000, while 42.2% earned above this threshold. It is important to note that the exchange rate for the US dollar during the study period ranged from approximately LBP 20,000 to 30,000 on the black market. When assessing food safety knowledge, over two-thirds (68.1%) of respondents rated their knowledge as “good”, while 16.6% rated it as “excellent” and 15.2% as “weak” (Table 1).

### 3.2. Basic Questions Related to Food Safety in Households

Questions were asked to investigate whether participants were food handlers and to understand their experiences with electricity outages (Table 2). Results revealed that 45.9% identified as primary food handlers, and 72.7% reported being involved in food preparation. Given Lebanon’s ongoing electricity issues, participants were asked whether they experienced outages at home. Only 6% reported no electricity outages, while 94% experienced at least two hours of power cuts daily. Of the 571 participants, 339 (59.4%) stated that they do not check the temperature of their refrigerators or freezers during the day. Regarding health-related issues, more than half of the respondents (54.1%) reported experiencing symptoms such as diarrhea, vomiting, fever, or abdominal pain in the past six months. Additionally, 65.3% acknowledged knowing someone who had suffered from food poisoning during the same period, and 13.8% reported being hospitalized due to food poisoning. In terms of meat consumption, 36% of participants preferred their meat cooked medium-rare or rare. However, 65.1% reported switching from ordering undercooked meat to well-done meat. Participants were also asked about their concerns regarding consuming food outside the home; 53.1% expressed a fear of eating certain types of non-home-prepared foods, with sushi as the most frequently mentioned (36.3%). Electricity outages also impacted food storage habits, as 74.3% of participants reported altering their methods of storing perishable foods due to refrigeration challenges during power cuts (Table 2).

### 3.3. Food Safety Knowledge Among Participants

The overall food safety knowledge score was calculated by summing all correct answers. The mean score was 10 ± 4.11 (<11, which represents 70% of the total score, ranging from 0 to 15), indicating insufficient food safety knowledge among participants in Lebanon. Over half of the respondents (52.7%) demonstrated poor food safety knowledge, while 47.3% achieved a good knowledge score. Table 3 presents some of the food safety knowledge questions used to calculate the score. For instance, 65.1% of participants incorrectly believed that food poisoning can occur only when contaminated food is consumed on the same day or the day before. Additionally, many respondents were unaware of the correct storage temperatures for freezers (56.7%) and refrigerators (51.3%). When asked more advanced questions about specific bacteria, a significant majority lacked knowledge. Specifically, 63.7%, 79.2%, 69.9%, and 64.3% of respondents were unfamiliar with *Escherichia coli*, *Campylobacter*, *Listeria*, and *Staphylococcus aureus*, respectively (Table 4). However, 64.3% of participants recognized *Salmonella*. It is important to note that the questions in Table 4 were not included in the overall food safety knowledge score.

### 3.4. Beliefs Towards the Risks Associated with Food Safety

The overall food safety belief score was calculated by summing all correct answers, as presented in Table 5, which includes some of the key questions. The mean score was 2.77 ± 1.37 (<4, representing 70% of the total belief score, ranging from 0 to 6), indicating unacceptable food safety beliefs among participants in Lebanon. A large majority (88.8%) demonstrated poor food safety beliefs, while only 64 participants (11.2%) achieved a good belief score. Findings from Table 5 showed that 55.2% of respondents were unaware of how long food remains safe in the refrigerator during an extended electricity outage. Only 44.8% correctly identified that food can remain safe for up to 4 h under such conditions. Additionally, 65.3% of participants believed that they could determine if food was adequately cooked based on subjective methods such as experience (smelling or tasting), visual appearance (color), or admitted they simply did not know (Table 5).

Results from Table 6 showed that 71.3% of the respondents limited their visits to restaurants because of a fear of getting food poisoning. Additionally, 57.1% of the participants strictly ate at home since they believed that food was stored safely. Moreover, 54.1% expressed the belief that being vegan or vegetarian now in Lebanon can limit exposure to food poisoning since some fruits and vegetables do not need to be refrigerated as much as meat and chicken (Table 6).

### 3.5. Participant Practices Associated with Food Safety

The mean food safety practices score was 10.79 ± 2.451 (<14, which is 70% of the total practice score ranging from 0 to 20), resulting in unacceptable food safety practices among the participants during electricity outages. The majority (93.9%) of participants (536 individuals) scored poorly, while only 6.1% (35 individuals) demonstrated good practices. Following the economic crisis and during ongoing electricity shortages, 82% of participants reported reducing their purchases of certain food items, including chicken, meat, fish, milk, and cheese (Table 7). Approximately 27.7% had to eat food that was not refrigerated properly because they had no food, and 37.7% ate inadequately refrigerated food because they did not like to throw it away. Moreover, only 20.3% of the respondents took the temperature of refrigerated or frozen food during an electricity outage (Table 7). Questions in Table 7 were included in the score except for the (after economic crises…) question.

**Figure 2:**
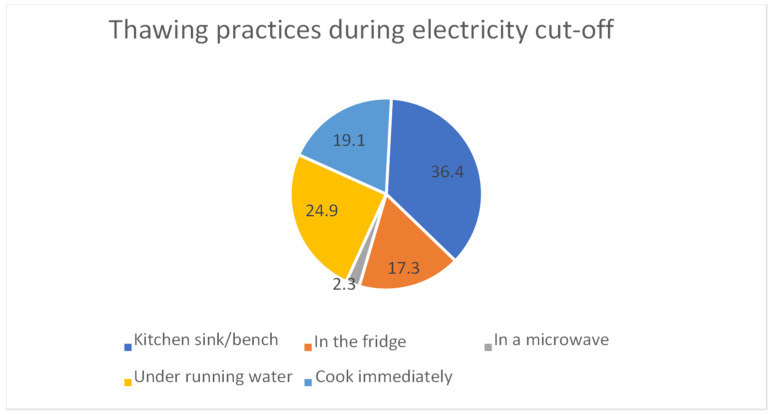
Distribution of thawing practices during electricity cut-off (%).

Different methods of food thawing were presented to the participants in order to determine the one that they usually follow in their households during electricity cut-off. A total of 36.4% of the samples thaw their frozen food on a kitchen bench or in a kitchen sink, whereas 63.6% illustrated good practices by choosing to thaw the food, whether inside the fridge, in a microwave, under running water, or to cook immediately (Figure 2).

Table 8 presents some of the asked practice questions during extended hours (more than 4 h). More than half (55.2%) of respondents chose to keep leftover cooked meals instead of discarding their meals, which is the correct action to do. In addition, 70.2% reported that they keep the refrigerated cut vegetables instead of discarding them. In contrast, 61.5% chose the right answer in discarding the refrigerated raw meat and chicken if the electricity was off for more than 4 h. Additionally, 72.3% decided to keep the refrigerated hard cheeses (cheddar, Swiss, parmesan) since it is the right option. Similarly, 62.7% chose the right action by discarding thawed meat and chicken without ice crystals inside the freezer.

### 3.6. Simple and Multiple Logistic Regression Analysis

Table 9 presents the results of simple and multiple logistic regression analyses examining the associations, socio-demographic characteristics, and the likelihood of having a good level of knowledge. In the simple logistic regression analysis, several variables were significantly associated with the likelihood of having a good level of knowledge in the study population. These included age (40–49: OR = 0.491, *p* = 0.010, 50–59: OR = 0.232, *p* < 0.001), governorate (South: OR = 0.333, *p* = 0.001, North: OR = 0.500, *p* = 0.039, Mount Lebanon: OR = 1.589, *p* = 0.021), nationality (OR = 0.471, *p* = 0.016), educational level (bachelor degree: OR = 1.592, *p* = 0.027, master/PhD: OR = 3.322, *p* = 0.001, technical school: OR = 4.948, *p* = 0.001), nationality (OR = 0.471, *p* = 0.016), household income (OR = 1.556, *p* = 0.045), the self-rating of food safety knowledge (Good: OR = 0.182, *p* < 0.001, Weak: OR = 0.033, *p* = 0.000), being the primary food handler in the household (OR = 1.409, *p* = 0.043), and the frequency of checking the temperature of fridges (Once/day: OR = 2.043, *p* = 0.001, Twice/day: OR = 3.075, *p* < 0.001, >3 times/day: OR = 9.969, *p* < 0.001). The multiple logistic regression model included the following variables: age, governorate, nationality, educational level, household income, self-rating of food safety knowledge, being the primary food handler, and frequency of checking the temperature of fridges. Results showed that participants aged 50–59 were less likely to achieve a good knowledge score compared to those aged 18–29 (OR = 0.242, CI: 0.094–0.621, *p* = 0.003). Moreover, residents of Mount Lebanon were more likely to achieve a good knowledge score compared to those in Beirut (OR = 1.889, CI: 1.201–2.974, *p* = 0.006). Regarding the educational level impact on the knowledge score, participants with master’s/PhD degrees (OR = 2.390, CI: 1.282–4.455, *p* = 0.006) or technical school education (OR = 3.069, CI: 1.012–9.310, *p* = 0.048) were more likely to score well compared to those with school-level education. Furthermore, participants who rated their knowledge as “Good” (OR = 0.228, CI: 0.112–0.463, *p* < 0.001) or “Weak” (OR = 0.049, CI: 0.020–0.121, *p* < 0.001) were less likely to achieve good scores compared to those who rated their knowledge as “Excellent”. Finally, checking the fridge temperature once per day (OR = 1.712, CI: 1.045–2.805, *p* = 0.033) or more than three times per day (OR = 5.135, CI: 1.803–14.625, *p* = 0.002) significantly increased the likelihood of achieving a good knowledge score compared to not checking at all.

Simple logistic regression analysis illustrated the associations of the socio-demographic characteristics with a positive belief score, showing that some variables that were significantly associated with the likelihood of having a positive belief included the educational level (bachelor degree: OR = 2.120, *p* = 0.013, master/PhD: OR = 2.635, *p* = 0.003), the self-rating of food safety knowledge (OR = 0.317, *p* = 0.009), the frequency of checking the temperature of the fridge/freezer (OR = 2.989, *p* < 0.001), and the total hours of electricity cut-off experienced per day in households (OR = 2.034, *p* = 0.010). Multiple logistic regression analysis also showed that respondents with a bachelor’s degree (OR = 2.104, CI: 1.139–3.886, *p* = 0.017) or a master’s/PhD (OR = 2.510, CI: 1.273–4.949, *p* = 0.008) were more likely to score positively compared to those with a school-level education. Regarding electricity outages, participants experiencing 2–4 h of daily electricity outages had higher odds of scoring positively compared to those with more than 4 h of outages (OR = 1.963, CI: 1.111–3.469, *p* = 0.020). As for checking fridge/freezer temperature, participants who checked the temperature twice per day (OR = 2.951, CI: 1.535–5.674, *p* = 0.001) were significantly more likely to achieve a positive belief score compared to those who did not check the temperature at all.

As shown in Table 9, simple logistic regression analysis also showed that a significant difference was observed between socio-demographic characteristics and the practice score. These variables included the total household income (OR = 1.855, *p* = 0.044), self-rating food safety knowledge (Good: OR = 0.487, *p* = 0.010, Weak: OR = 0.180, *p* = 0.001), and the frequency of checking the temperature of the fridge/freezer (Once/day: OR = 2.515, *p* = 0.002, Twice/day: OR = 4.226, *p* < 0.001, and more than three times/day: OR = 4.677, *p* < 0.001). In the multiple logistic regression analysis, checking the temperature of fridges and freezers was significantly associated with improved food safety practices. Participants who checked the temperature once per day (OR = 2.177, CI: 1.193–3.972, *p* = 0.011), twice per day (OR = 3.247, CI: 1.576–6.692, *p* = 0.001), or more than three times per day (OR = 3.437, CI: 1.579–7.480, *p* = 0.002) had higher odds of exhibiting better food safety practices compared to those who did not check the temperature at all.

### 3.7. Association Between the Different KAP Scores

Table 10 shows significant associations between participants’ knowledge, beliefs, and practices. Notably, a significant relationship exists between respondents’ beliefs and their practices. The odds of exhibiting poor practices were 1.746 times higher among participants with insufficient knowledge compared to those with good knowledge. Furthermore, poor beliefs were more likely to occur among participants with inadequate food safety knowledge, with an odds ratio (OR) of 2.167, compared to those with sufficient knowledge. Additionally, poor beliefs were more likely among participants with poor food safety practices, with an OR of 2.061, compared to those with good practices.

## 4. Discussion

Throughout the years, food safety has reached a wider range of people than it ever did. Despite all the knowledge that people acquired in the field of food safety and the major attempts from all concerned stakeholders, there is still a major gap due to the insufficiency of awareness among populations regarding the safety of food. Especially, food can be contaminated at any stage of the food chain from receiving to consumption and can result in a serious risk of foodborne diseases [2]. In the present study, the knowledge, beliefs, and practices of consumers in households regarding food safety during electricity outages were assessed.

The overall food safety knowledge was unsatisfactory (mean score was 10 ± 4.112 < 11). Similarly, very poor food safety knowledge among food handlers was reported in households in China [30]. In the current study, results showed that 56.7% answered wrongly or did not know the temperature of frozen food (Refer to Table 3). A study conducted in South Africa noted that 72% of the participants had no clue about the proper temperature of freezers [31]. Regarding the knowledge of refrigerator temperature, our results found that 51.3% of the participants did not know the correct range of temperature (Refer to Table 3). Another study conducted by Jevsnik et al., (2007) found that 56.3% of the consumers reported knowing the proper temperature of the fridges [32]. Moreover, 59.5% reported that freezing is not effective in killing bacteria and viruses (refer to Table 3). This rate was higher than a study conducted also in Lebanon in which only 35.8% knew that [14].

In a previous study conducted by Faour-Klingbeil (2016), 77.5% of food handlers took the taste and smell as a reference in determining if the food was contaminated [33], whereas, in this study, a lower percentage was found, since 41.7% believed the same (refer to Table 3). However, this subject is very important among populations since the safety of food cannot be determined by the sensory properties, because some pathogens do not change the appearance nor the taste of contaminated food and still can cause food poisoning if consumed [34].

More than half (65.1%) of the participants agreed that food poisoning can happen as a result of consuming contaminated food only if it is consumed the same day or the day before (refer to Table 3). This can also be an indicator of a knowledge gap among the subjects since the onset of food poisoning symptoms can vary depending on the type of the pathogen, and it can take hours, days, or even weeks to appear [35].

Regarding the beliefs towards food safety, the overall score was poor (mean score was 2.77 ± 1.372 < 4) with 71.8% of the participants tending to purchase high-risk food (dairy, meat, chicken…) from big chain supermarkets instead of small local groceries because they believe that they are safer (refer to Table 6). However, there is no proof that chain supermarkets do not experience a shortage of electricity throughout the day and especially at night. Jevsnik et al., (2007) stated that 67.8% of the respondents do not check the temperature in retail markets since it was the least important factor that the customers cared about. They explained this by the tendency of consumers to trust big chain supermarkets [32]. According to the Centers for Disease Control and Prevention, the safest way to cook food is to reach the required temperature to kill pathogens that cause illnesses [36]. In this study, 65.3% depended on their experience (smell and taste of the food) or visual appearance (color of food) to decide if the food was cooked enough (refer to Table 5). A higher rate (78.31%) judging the doneness of food by the senses was observed in a study conducted in Saudi Arabia [37]. Moreover, 54.1% of the subjects believed that being vegan or vegetarian in Lebanon would reduce the chance of getting food poisoning since some vegetables do not need to be refrigerated as much as meat and chicken (refer to Table 6). In fact, according to the Australian Institute of Food Safety (2019), people who follow vegan or vegetarian diets can also get foodborne illnesses. Some have the misconception of linking the poisoning only to meat, seafood, and cheese, but plant-based food can easily be contaminated with toxins that can naturally occur, such as parasites, viruses, and bacteria, at any stage, starting from the farm [38].

The total food safety practices during the electricity outage was unacceptable (mean score 10.79 ± 2.451 < 14). Our findings indicated that 36.4% of the participants thaw their frozen food on a kitchen bench or in the kitchen sink (refer to Figure 1). Similar results were reported in South Africa since 28% used their kitchen surfaces for thawing the food, and they also confirmed the contamination of kitchen surfaces with different pathogens such as *Listeria*, *Salmonella* spp. and *Escherichia coli*. These findings highlight the danger of using unsafe methods to thaw food [31]. However, Jevsnik et al., (2007) reported a higher rate (50.4%) of participants who thaw frozen food on a kitchen bench [32].

After preparing and cooking the food that will be eaten 3–4 h later, we found that 57.8% of the participants stored the food at room temperature and then reheated it before consumption (Refer to Table 8). The findings of this study were higher when compared to other similar studies. To illustrate, a study in Lebanon reported that 45.5% keep their food at room temperature whether on the counter or inside the oven [14]. In Slovenia, they found that only 12.5% leave their food at room temperature until they are eaten [32]. This emphasizes the differences between food safety practices between different populations and countries. According to the Food and Drug Administration, food should not be left at room temperature for more than 2 h because bacteria can grow rapidly and can reach levels where food, if consumed, can cause illness [39]. During an extended electricity outage, 62.7% discarded thawed frozen food that had no ice crystals. A total of 61.5% discarded refrigerated meat/chicken, and 45.9% discarded refrigerated soft cheeses after 4 h of electricity cut-off (refer to Table 8). Meanwhile, in the USA, when the same questions were asked, only 37.1% threw thawed food inside the freezer, and only 33% knew that they should throw refrigerated perishable food after 4 h of power outage [22]. Moreover, our results showed that only 20.3% of the participants take the temperature of the refrigerated food during electricity shortages (refer to Table 7). Kosa et al., (2011) reported an even lower rate (9%) did that [22].

According to the Lebanon Food Security Portal (25), which was developed by the American University of Beirut, inflation rates, dollarization, and the war in Ukraine caused a huge increase in the costs of food by more than 400% between February 2021 and February 2022. This issue is affecting both the availability and the accessibility of food [40]. This might explain why 82% of the respondents of this study reduced their purchasing of meat, chicken, dairy, and fish. A total of 27.7% also had to eat food that was not refrigerated properly since they had no other choice (refer to Table 7).

As demonstrated in the present study, it is important to mention that good food safety knowledge reflects good food safety practices and vice versa. The opposite was observed in a similar study in Lebanon among married women where they found that the knowledge of food safety may not indicate proper food safety practices [25]. Another article assessed food safety knowledge, attitudes, and practices among food handlers in Malaysia, and they concluded that, despite scoring good food safety knowledge, improper and unsafe practices were performed while handling the food [41].

## 5. Limitations

Limitations of this study should be declared. A key limitation is that data collection was conducted online, preventing control over participant distribution. Relying on social media and online platforms introduces the risk of selection bias, as users of these platforms may not be representative of the broader population. For instance, most respondents were from Mount Lebanon, with limited participation from other regions such as the North, South, and Bekaa. This uneven geographic distribution may limit the generalizability of the findings, particularly in capturing the knowledge and practices of individuals outside the Mount Lebanon area. Another limitation is the overrepresentation of younger participants, with the majority of respondents aged 18–29. This may skew the results, as younger individuals may have different knowledge and behaviors related to food safety compared to older generations. Additionally, more than half of the participants held at least a bachelor’s degree, which could influence findings, as higher education levels are often associated with greater awareness of food safety risks and access to relevant information. This demographic imbalance may limit this study’s ability to fully represent the broader population, particularly individuals with lower education levels or older adults. Furthermore, participation was restricted to individuals with internet access, excluding those without it. This exclusion may affect the diversity of respondents, as individuals without internet access could belong to different socioeconomic groups or regions, potentially resulting in an incomplete representation of food safety knowledge across Lebanon. Lastly, the self-reported nature of the data introduces the possibility of information bias. Participants may have responded in ways they perceived as socially acceptable or desirable, particularly on food safety-related questions, leading to social desirability bias. Additionally, variations in how participants understood and interpreted the questions could have resulted in inconsistencies in responses, further contributing to potential bias in the findings. Overall, these limitations underscore the need for future studies to adopt diverse data collection methods, ensure a more representative sample, and implement strategies to minimize biases. Doing so will enhance the reliability and generalizability of findings.

## 6. Conclusions

To our knowledge, the present research is the first study in Lebanon and the Middle East that presents data concerning food safety knowledge, beliefs, and practices during electricity cut-off, highlighting many misconceptions about food safety. This current study provides a baseline to conduct further analysis to understand the situation in Lebanon. As the first study of its kind in the region, this research establishes a valuable baseline for future comparisons, enabling the monitoring of changes or improvements in food safety practices and awareness over time. Its significance lies in identifying gaps in food safety knowledge and practices during electricity outages—an issue of growing concern in Lebanon. Notably, the findings highlight widespread misconceptions about food safety, with many individuals unaware of the risks associated with power disruptions and how they can lead to food contamination. The findings urge the governmental and nongovernmental authorities to take actions to enhance food safety in Lebanon, given the alarming state of food safety and security. Moreover, national campaigns should be carried on to increase awareness and improve the knowledge among Lebanese populations about food safety practices during electricity outages. More frequent inspections are to be performed to ensure that food safety regulations are not violated in retail shops to keep the food safe for consumption.

## Figures and Tables

**Table 1:** Sociodemographic characteristics of participants (n = 571).

Characteristics		Frequency (n) Percentage (%)
Age	18–29 30–39 40–49 50–59 60 and more	300 (52.5%) 156 (27.3%) 65 (11.4%) 38 (6.7%) 12 (2.1%)
Gender Marital Status Governorate of Lebanon Nationality Educational Level Total Household Income How Do You Rate Your Food Safety Knowledge?	Female Male Single Married Divorced, Widowed, and Separated Mount Lebanon Beirut South North Bekaa Lebanese Non-Lebanese School Certificate University Bachelor Master/PhD Technical School Less than LBP 1,000,000 LBP 1,000,000–5,000,000 LBP 5,000,000–10,000,000 More than LBP 10,000,000 Excellent Good Weak	348 (60.9%) 223 (39.1%) 328 (57.4%) 222 (38.9%) 21 (3.8%) 279 (48.9%) 165 (28.9%) 56 (9.8%) 48 (8.4%) 23 (4.0%) 524 (91.8%) 47 (8.2%) 140 (24.5%) 270 (47.3%) 134 (23.5%) 27 (4.7%) 13 (2.3%) 131 (22.9%) 186 (32.6%) 241 (42.2%) 95 (16.6%) 389 (68.1%) 87 (15.2%)

**Table 2:** Basic questions related to food safety during electricity cut-off.

Question Statement	Variables	Frequency (n) Percentage (%)
Are you the primary food handler in your household?	Yes No	262 (45.9%) 309 (54.1%)
Are you involved in food preparation at your house?	Yes No	415 (72.7%) 156 (27.3%)
How many hours per day do you experience electricity cut-off at your house?	I don’t experience electricity cut-off Less than 2 h 2–4 h More than 4 h	34 (6.0%) 23 (4.0%) 76 (13.3% 438 (76.7%)
How often do you check the temperature of your fridge/freezer?	Once/day Twice/day More than 3 times/day I don’t check it	126 (22.1%) 60 (10.5%) 46 (8.1%) 339 (59.4%)
Did you experience diarrhea, vomiting, fever, or abdominal pain in the past 6 months?	Yes No	309 (54.1%) 262 (45.9%)
Have you been hospitalized because of food poisoning in the past 6 months?	Yes No	79 (13.8%) 492 (86.2%)
Do you know anyone (other than yourself) who got food poisoning in the past 6 months?	Yes No	373 (65.3%) 198 (34.7%)
How do you usually eat your meat?	Well-done Medium-rare Rare I don’t eat meat	342 (59.9%) 167 (29.2%) 39 (6.8%) 23 (4.0%)
With the electricity cutoff and with the increase in food poisoning cases in Lebanon, did you shift from ordering medium-rare meat to order well-done meat?	Yes No	372 (65.1%) 199 (34.9%)
What food are you afraid the most to eat from outside your house (restaurant) during the electricity cut-off?	Burgers/sandwiches Sushi Salads Everything Nothing	49 (8.6%) 207 (36.3%) 47 (8.2%) 182 (31.9%) 86 (15.1%)
Did the electricity cuts change your perishable food (foods that need a refrigerator: meat, chicken, dairy) storage habits?	Yes No	424 (74.3%) 147 (25.7%)

**Table 3:** Score distribution of food safety knowledge questions.

Question Statement	Correct Answer	Wrong Answer
Do you know that foodborne pathogens can multiply on food that was not refrigerated? Food poisoning can happen as a result of consuming contaminated food on the same day or the day before only If the smell and color of food seem okay, that means the food is not contaminated Storing raw chicken in the fridge without proper precaution can contaminate other food What is the optimal temperature of frozen food? What is the optimal temperature of fridge? Is freezing enough to eliminate foodborne bacteria and viruses? Choose the best way to reduce the risk of contaminated food (cooking, washing the food, refrigeration, I don’t know)	69.9% (399) 34.9% (199) 58.3% (333) 71.5% (408) 43.3% (247) 48.7% (278) 59.5% (340) 59.2% (338)	30.1% (172) 65.1% (372) 41.7% (238) 28.5% (163) 56.7% (324) 51.3% (293) 40.5% (231) 40.8% (233)

**Table 4:** Knowledge regarding different bacteria.

Question Statement	Response	Frequency (n) Percentage (%)
Do you know what *Escherichia coli* is? Do you know what *Campylobacter* is? Do you know what *Listeria* is? Do you know what *Salmonella* is? Do you know what *Staphylococcus aureus* is?	Yes No Yes No Yes No Yes No Yes No	207 (36.3%) 364 (63.7%) 119 (20.8%) 452 (79.2%) 172 (30.1%) 399 (69.9%) 479 (83.9%) 92 (16.1%) 204 (35.7%) 367 (64.3%)

**Table 5:** Score distribution of food safety belief questions.

Question Statement	Correct Answer	Wrong Answer
During a long electricity cut-off, for how long do you think the fridge will keep the food safely cool? During a long electricity cut-off, for how long do you think a full-packed freezer will keep the food safely frozen? During a long electricity cut-off, for how long do you think a half-packed freezer will keep the food safely frozen? How do you know if food is cooked enough?	44.8% (256) 25.2% (144) 46.2% (264) 34.7% (198)	55.2 (315) 74.8% (427) 53.8% (307) 65.3% (373)

**Table 6:** Beliefs towards eating/purchasing habits during electricity cut-off.

Question Statement	Response	Frequency (n) Percentage (%)
During the electricity crises, did you limit your visits to restaurants for fear of getting food poisoning?	Yes No	407 (71.3%) 164 (28.7%)
During the electricity crises, did you strictly eat at home because you know that food has been safely stored (frozen/refrigerated)?	Yes No	326 (57.1%) 245 (42.9%)
In the electricity crises, for purchasing high-risk food (dairy, meat, chicken, fish), do you use big chain supermarkets instead of small local grocery stores since they are safer?	Yes No	410 (71.8%) 161 (28.2%)
Do you believe that being vegan or vegetarian now in Lebanon will reduce the chance of getting food poisoning?	Yes No	309 (54.1%) 262 (45.9%)

**Table 7:** Economic crises along with electricity cut-off effect on purchasing/eating practices.

Question Statement	Response	Frequency (n) Percentage (%)
After the economic crisis and electricity shortages, did you reduce your purchasing of certain food (meat, chicken, fish, cheese, milk)? Have you ever eaten food that was not refrigerated properly because you had no other food? Have you ever eaten food that was not refrigerated properly because you don’t like to throw food away? During electricity cut-off, do you take the temperature of food inside fridge/freezer?	Yes No Yes No Yes No Yes No	468 (82%) 103 (18%) 158 (27.7%) 413 (72.3%) 215 (37.7%) 356 (62.3%) 116 (20.3%) 455 (79.7%)

**Table 8:** Score distribution of food safety practice questions.

Question Statement	Correct Answer	Wrong Answer
After preparing and cooking the food that you will eat 3–4 h later, what do you usually do? During extended electricity cut-off (more than 4 h), do you discard or keep leftover cooked meals inside fridge? During extended electricity cut-off (more than 4 h), do you discard or keep raw chicken/meat inside fridge? During extended electricity cut-off (more than 4 h), do you discard or keep soft cheeses inside fridge? During extended electricity cut-off (more than 4 h), do you discard or keep hard cheeses inside fridge? During extended electricity cut-off (more than 4 h), do you discard or keep cut vegetables inside fridge? During extended electricity cut-off (more than 4 h), do you discard or keep uncut vegetables inside fridge? During extended electricity cut-off (more than 4 h), do you discard or keep thawed meat/chicken without ice crystals inside freezer?	42.2% (241) 44.8% (256) 61.5% (351) 45.9% (262) 72.3% (413) 29.8% (170) 94.6% (540) 62.7% (358)	57.8 (330) 55.2% (315) 38.5% (220) 54.1% (309) 27.7% (158) 70.2% (401) 5.4% (31) 37.3% (213)

**Table 9:** Simple and multiple logistic regression for the association of characteristics of the study population with levels of food safety knowledge, belief, and practice (n = 571).

	Knowledge		Belief		Practice	
	Simple Logistic Regression OR, (95% CI), *p*-Value	Multiple Logistic Regression OR, (95% CI), *p*-Value	Simple Logistic Regression OR, (95% CI), *p*-Value	Multiple Logistic Regression OR, (95% CI), *p*-Value	Simple Logistic Regression OR, (95% CI), *p*-Value	Multiple Logistic Regression OR, (95% CI), *p*-Value
Gender Female Male	1 1.217 (0.867, 1.710), *p* = 0.256		1 0.823 (0.536, 1.264), *p* = 0.374		1 0.899 (0.557, 1.451), *p* = 0.662	
Age 18–29 30–39 40–49 50–59 60+	1 0.884 (0.597, 1.310), *p* = 0.539 0.491 (0.285, 0.844), *p* = 0.010 * 0.232 (0.108, 0.494), *p* < 0.001 * 0.648 (0.204, 2.058), *p* = 0.462	1 0.993 (0.602, 1.639), *p* = 0.980 0.579 (0.292, 1.147), *p* = 0.117 0.242 (0.094, 0.621), *p* = 0.003 * 0.725 (0.199,2.634), *p* = 0.625	1 1.067 (0.669, 1.701), *p* = 0.786 0.518 (0.235, 1.140), *p* = 0.102 0.691 (0.277, 1.726), *p* = 0.429 0.000 (0.000), *p* = 0.999		1 0.608 (0.342, 1.079) *p* = 0.089 0.749 (0.349, 1.608) *p* = 0.458 0.259 (0.060, 1.109) *p* = 0.069 0.932, (0.198, 4.378) *p* = 0.929	
Governorate of Lebanon Beirut South North Mount Lebanon Bekaa	1 0.333 (0.173, 0.642), *p* = 0.001 * 0.500 (0.258, 0.967), *p* = 0.039 * 1.589 (1.072, 2.355), *p* = 0.021 * 0.536 (0.220, 1.307), *p* = 0.170	1 0.588 (0.277, 1.249), *p* = 0.167 0.542 (0.240, 1.221), *p* = 0.139 1.889 (1.201, 2.974), *p* = 0.006 * 0.640 (0.226, 1.811), *p* = 0.400	1 0.693 (0.298, 1.608), *p* = 0.393 0.831 (0.355, 1.948), *p* = 0.671 1.212 (0.752, 1.954), *p* = 0.429 0.396 (0.088, 1.776), *p* = 0.226		1 0.605 (0.219, 1.676), *p* = 0.334 1.625 (0.713, 3.704), *p* = 0.248 1.125 (0.651, 1.945), *p* = 0.673 0.926 (0.255,3.368), *p* = 0.907	
Educational level School Bachelor’s Degree Master/PhD Technical School Nationality Lebanese Non-Lebanese	1 1.592 (1.054, 2.405), *p* = 0.027 * 3.322 (2.015, 5.478), *p* < 0.001 * 4.948 (1.880, 13.022), *p* = 0.001 * 1 0.471 (0.255, 0.869), *p* = 0.016 *	1 1.107 (0.662, 1.851), *p* = 0.699 2.390 (1.282, 4.455), *p* = 0.006 * 3.069 (1.012, 9.310), *p* = 0.048 * 1 0.733 (0.351, 1.533), *p* = 0.409	1 2.120 (1.168, 3.849), *p* = 0.013 * 2.635 (1.376, 5.047), *p* = 0.003 * 1.761 (0.585, 5.301), *p* = 0.314 1 1.264 (0.622, 2.569), *p* = 0.517	1 2.104 (1.139, 3.886), *p* = 0.017 * 2.510 (1.273, 4.949), *p* = 0.008 * 1.396 (0.447, 4.361), *p* = 0.566	1 0.783 (0.443, 1.382), *p* = 0.398 0.892 (0.465, 1.714), *p* = 0.732 1.156 (0.397, 3.367), *p* = 0.790 1 0.837 (0.344, 2.037), *p* = 0.695	
Household Income >LBP 10,000,000 LBP 5,000,000–10,000,000 LBP 1,000,000–5,000,000 <LBP 1000,000	1 1.404 (0.954, 2.065), *p* = 0.085 1.556 (1.009, 2.398), *p* = 0.045 * 0.850 (0.278, 2.603), *p* = 0.776	1 1.212 (0.745, 1.972), *p* = 0.439 1.024 (0.593, 1.767) *p* = 0.932 0.764 (0.199, 2.940), *p* = 0.696	1 1.340 (0.829, 2.166), *p* = 0.233 1.173 (0.681, 2.020), *p* = *0.564 2.106* (0.619, 7.161), *p* = 0.233		1 1.718 (0.984, 3.000), *p* = 0.057 1.855 (1.017, 3.384), *p* = 0.044 * 1.503 (0.316, 7.159), *p* = 0.609	1 1.344 (0.748, 2.417), *p* = 0.323 1.276 (0.677, 2.406), *p* = 0.451 1.311 (0.265, 6.489), *p* = 0.740
Knowledge Rating Excellent Good Weak	1 0.182 (0.096, 0.345), *p* < 0.001 * 0.033 (0.014, 0.073), *p* < 0.001 *	1 0.228 (0.112, 0.463), *p* < 0.001 * 0.049 (0.020, 0.121), *p* < 0.001	1 0.836 (0.493, 1.419), *p* = 0.507 0.317 (0.133, 0.753), *p* = 0.009 *	1 1.258 (0.705,2.247), *p* = 0.437 0.582 (0.229, 1.481), *p* = 0.256	1 0.487 (0.283, 0.839), *p* = 0.010 * 0.180 (0.065, 0.498), *p* = 0.001 *	1 0.711 (0.397, 1.275), *p* = 0.253 0.350 (0.120, 1.022), *p* = 0.055
Are You The Primary Food Handler In Your Household? Yes No Checking the Temperature of Fridge/Freezer I don’t check it Once/day Twice/day >3 times/day Total Hours Of Electricity Cut-Off Experienced Per Day In Households: >4 h 2–4 h <2 h I Don’t Experience Electricity Cut-off	1 1.409 (1.011, 1.963), *p* = 0.043 * 1 2.043 (1.343, 3.109), *p* = 0.001 * 3.075 (1.686, 5.608), *p* < 0.001 * 9.969 (3.845, 25.847), *p* < 0.001 * -	1 1.296 (0.837, 2.008), *p* = 0.246 1 1.712 (1.045, 2.805), *p* = 0.033 * 1.539 (0.763, 3.106), *p* = 0.229 5.135 (1.803, 14.625), *p* = 0.002 * -	1 0.993 (0.657, 1.501), *p* = 0.975 1 1.278 (0.756, 2.159), *p* = 0.359 2.989 (1.642, 5.443), *p* < 0.001 * 1.623 (0.777, 3.389), *p* = 0.198 **1** 2.034 (1.185, 3.492), *p* = 0.010 * 0.420 (0.096, 1.826), *p* = 0.247 0.944 (0.379, 2.356), *p* = 0.902	1 1.191 (0.693, 2.049), *p* = 0.527 2.951 (1.535, 5.674), *p* = 0.001 * 2.095 (0.925, 4.746), *p* = 0.076 **1** 1.963 (1.111, 3.469), *p* = 0.020 * 0.342 (0.077, 1.526), *p* = 0.160 0.912 (0.360,2.315), *p* = 0.847	1 0.975 (0.613, 1.551), *p* = 0.914 1 2.515 (1.401, 4.516), *p* = 0.002 * 4.226 (2.145, 8.328), *p* < 0.001 * 4.677 (2.244, 9.747), *p* < 0.001 * -	1 2.177 (1.193, 3.972), *p* = 0.011 * 3.247 (1.576, 6.692), *p* = 0.001 * 3.437 (1.579, 7.480), *p* = 0.002 *

* Estimates shown in bold are those that are statistically significant at *p* < 0.05.

**Table 10:** Association between participants’ knowledge and their beliefs and practices.

	Total (n = 571)	Poor Practices n (%)	Good Practices n (%)	Significance; OR (CI)	Poor Beliefs n (%)	Good Beliefs n (%)	Significance; OR (CI)
Bad Knowledge	255	227 (89%)	28 (11%)	0.024 *; **1.746 (1.073; 2.843)**	221 (86.7%)	34 (13.3%)	0.001 *; **2.167 (1.393; 3.37)**
Good Knowledge	316	260 (82.3%)	56 (17.7%)		237 (75%)	79 (25%)	
Bad Practices	487				400 (82.1%)	87 (17.9%)	0.005 *; **2.061 (1.228; 3.458)**
Good Practices	84				58 (69%)	26 (31%)	

* Estimates shown in bold are those that are statistically significant at *p* < 0.05.

## Data Availability

The data presented in this study are available upon request from the corresponding author. The data are not publicly available due to the privacy of participants and ethical concerns.

## Supplementary Materials

The following supporting information can be downloaded at: https://www.mdpi.com/article/10.3390/foods14050855/s1, Supplementary File S1: Invitation script; Supplementary File S2: Consent form, Supplementary File S3: Survey; Supplementary File S4: Chi-square test of independence
