# Supplementary material for: Beliefs, Practices, and Knowledge of Household Food Handlers Regarding the Impact of Electricity Outages on Food Safety: Findings from a National Cross-Sectional Study in Lebanon
Source: Foods. 2025 Mar 2;14(5):855. doi: 10.3390/foods14050855 (PMC11898753; doi:10.3390/foods14050855)
Supplement: Supplementary file 1 [file foods-14-00855-s001.zip › Supplementary Material File S1.pdf]

AUB Social & Behavioral Sciences

INVITATION SCRIPT

Invitation to Participate in a Research Study

This notice is for an AUB-IRB Approved Research Study for Dr. Samer Kharroubi at AUB. (Phone: (01) 350 000 Ext: 4541)

(Email: sk157@aub.edu.lb)

It is not an Official Message from AUB

I am inviting you to participate in a research study about" Beliefs, practices and knowledge of food handlers in households regarding electricity outage effect on food safety: National cross-sectional study in Lebanon."

You will be asked to complete a short survey/questionnaire with demographic information

You are invited because we are targeting people who are at least 18 years old and are currently residing in Lebanon

The estimated time to complete this survey is approximately 10 minutes

The research is conducted online and is hosted on AUB server

Please read the consent form and consider whether you want to be involved in the study

If you have any questions about this study, you may contact the investigator/research team (Noura Abou Assaly, 70920682, noa13@mail.aub.edu)

Institutional Review Board
American University of Beirut

16 FEB 2022

APPROVED
