# Supplementary material for: Beliefs, Practices, and Knowledge of Household Food Handlers Regarding the Impact of Electricity Outages on Food Safety: Findings from a National Cross-Sectional Study in Lebanon
Source: Foods. 2025 Mar 2;14(5):855. doi: 10.3390/foods14050855 (PMC11898753; doi:10.3390/foods14050855)
Supplement: Supplementary file 1 [file foods-14-00855-s001.zip › Supplementary Material File S3.pdf]

SURVEY

Beliefs, practices and knowledge of food handlers in households regarding electricity outage effect on food safety: National cross-sectional study in Lebanon

Principle Investigator: Dr. Samer Kharroubi

Student: Noura Abou Assaly

Institutional Review Board
American University of Beirut

16 FEB 2022

APPROVED

Section 1: Socio - demographic characteristics

1. Please select your age group:
    a) (18 – 29)
    b) (30 – 39)
    c) (40 – 49)
    d) (50 – 59)
    e) (60 and above)

2. What is your gender?
    a) Male
    b) Female
    c) Other

3. What is your marital status?
    a) Single
    b) Married
    c) Divorced
    d) Widowed
    e) Separated

4. In which governorate of Lebanon do you live?
    a) Beirut
    b) South
    c) North
    d) Mount Lebanon
    e) Bekaa

5. What is your nationality?
    a) Lebanese
    b) Non-Lebanese. Please specify ______

6. What is your highest educational level achieved?
    a) Primary school

Institutional Review Board
American University of Beirut

16 FEB 2022

APPROVED

b) Middle school
c) High school
d) University degree (Bachelor)
e) University degree (Masters/PhD)
f) Technical school

7. If you choose a university degree, please specify the major _________

8. What is the total monthly income of your household?
   a) Less than 1,000,000 L.L
   b) 1,000,000 – 5,000,000 L.L
   c) 5,000,000 – 10,000,000 L.L
   d) More than 10,000,000 L.L

Section 2: Basic questions related to food safety in households

1. Are you the primary food handler in your household?
   a) Yes
   b) No

2. Are you involved in food preparation at your house?
   a) Yes
   b) No

3. How many hours per day do you experience electricity cut off at your house?
   a) I don't experience electricity cut off
   b) Less than 2 hours
   c) 2 – 4 hours
   d) More than 4 hours

4. If you experience an electricity cutoff, can you specify the schedule of electricity cutoff at your house?
   a) Not more than 2 hours continuous
   b) Between 2 – 4 hours continuous
   c) More than 4 hours continuous

5. With electricity cuts, are you spending more effort on cooking food (using oven, grill, stove) that you usually consume directly from the fridge (e.g. Deli meat, cheese...)?

Institutional Review Board
American University of Beirut

16 FEB 2022

APPROVED

a) Yes
b) No

6. Did the electricity cuts change your perishable food (foods that need refrigerator: meat, chicken, dairy) storage habits?
   a) Yes
   b) No

7. With the electricity cuts, did you cut down on purchasing perishable foods due to the inability to refrigerate properly?
   a) Yes
   b) No

8. Do you know that deficient refrigeration may affect the safety and quality of food?
   a) Yes
   b) No

9. Do you know that foodborne pathogens can survive refrigeration?
   a) Yes
   b) No

10. Do you know that foodborne pathogens can multiply on food that was not refrigerated properly?
    a) Yes
    b) No

11. Did you experience diarrhea, vomiting, fever, or abdominal pain in the past 6 months?
    a) Yes
    b) No

12. Have you been hospitalized because of food poisoning in the past 6 months?
    a) Yes
    b) No

13. If yes, did the doctors confirm that it's food poisoning?
    a) Yes
    b) No

Institutional Review Board
American University of Beirut

16 FEB 2022

APPROVED

14. Do you know anyone (other than yourself) who got food poisoning in the past 6 months?
    a) Yes
    b) No

15. Do you know anyone (other than yourself) who experienced diarrhea, vomiting, fever, or abdominal pain in the past 6 months?
    a) Yes
    b) No

16. Have you ever eaten food that was not refrigerated properly because you had no other food?
    a) Yes
    b) No

17. Have you ever eaten food that was not refrigerated properly because you don't like to throw food?
    a) Yes
    b) No

18. Do you know what Escherichia coli is?
    a) Yes
    b) No

19. Do you know what Campylobacter is?
    a) Yes
    b) No

20. Do you know what Listeria is?
    a) Yes
    b) No

21. Do you know what Salmonella is?
    a) Yes
    b) No

22. Do you know what Staphylococcus aureus is?

Institutional Review Board
American University of Beirut

16 FEB 2022

APPROVED

a) Yes
b) No

23. Do you know that food can be contaminated by bacteria that can make you sick?
    a) Yes
    b) No

24. Do you know that food can be contaminated by viruses that can make you sick?
    a) Yes
    b) No

25. Do you know that food can be contaminated by parasites that can make you sick?
    a) Yes
    b) No

26. Choose the best way to reduce the risk of contaminated food among the listed?
    a) Cooking
    b) Refrigeration
    c) Washing the food
    d) I don't know

27. How do you rate your food safety knowledge?
    a) Excellent
    b) Good
    c) Weak

Section 3: Knowledge about food safety

1. Food poisoning can happen as a result of consuming contaminated food on the same day or the day before only.
    a) True
    b) False
    c) I don't know

2. If the smell and the color of the food seem okay, that means that the food is not contaminated.
    a) True

Institutional Review Board
American University of Beirut

16 FEB 2022

APPROVED

b) False
c) I don’t know

3. Storing raw chicken in the fridge without proper precaution can contaminate other food in the fridge.
   a) False
   b) True
   c) I don’t know

4. Drippings from raw chicken and meat can contaminate food in the fridge and kitchen.
   a) True
   b) False
   c) I don’t know

5. How do you thaw frozen foods?
   a) On the kitchen bench
   b) In the kitchen sink
   c) In the fridge
   d) In a microwave
   e) Under running water
   f) Cook immediately

6. How do you thaw frozen foods during extended electricity shortages?
   a) On the kitchen bench
   b) In the kitchen sink
   c) In the fridge
   d) In a microwave
   e) Under running water
   f) Cook immediately

7. What is the optimal temperature of frozen food?
   a) 0°c
   b) - 5°c
   c) - 18°c
   d) I don’t know

8. What is the optimal temperature of fridge?

Institutional Review Board
American University of Beirut

16 FEB 2022

APPROVED

a) 1 - 4°c
b) 5 - 9°c
c) 10 - 12°c
d) I don't know

9. Is freezing enough to eliminate foodborne bacteria and viruses?
   a) Yes
   b) No

10. Is refrigeration enough to eliminate foodborne bacteria and viruses?
    a) Yes
    b) No

11. In the electricity crisis, for purchasing high-risk food (dairy, meat, chicken, fish..), do you use big chain supermarkets instead of small local grocery stores since they are safer?
    a) Yes
    b) No

12. After preparing and cooking the food that you will eat 3 – 4 hours later. What do you usually do?
    a) Store the food at room temperature then reheat it
    b) Store the food inside microwave/oven then reheat it
    c) Store the food inside the fridge then reheat it

13. How do you know if food is cooked enough?
    a) By experience (smelling and tasting the food)
    b) By cooking according to the recommended time to the required temperature
    c) By visual appearance (color of the food)
    d) I don't know

14. During a long electricity cutoff, for how long do you think the fridge will keep the food safely cool (if doors are kept closed)?
    a) 4 hours
    b) 5 - 12 hours
    c) More than 12 hours
    d) I don't know

Institutional Review Board
American University of Beirut

16 FEB 2022

APPROVED

15. During a long electricity cutoff, for how long do you think a full packed freezer will keep the food safely frozen?
    a) 24 hours
    b) 48 hours
    c) 72 hours
    d) I don't know

16. During a long electricity cutoff, for how long do you think a half packed freezer will keep the food safely frozen?
    a) 24 hours
    b) 48 hours
    c) 72 hours
    d) I don't know

Section 4: Beliefs towards the risks associated with food safety

1. During the electricity crisis, did you limit your visits to restaurants for fear of getting food poisoning?
   a) Yes
   b) No

2. During the electricity crisis, did you strictly eat at home because you know that the food has been safely stored (frozen/cold)?
   a) Yes
   b) No

3. Do you believe that being vegan or vegetarian now in Lebanon will reduce the chance of food poisoning? Since some vegetables don't need to be refrigerated as much as meat and chicken.
   a) Yes
   b) No

4. How do you usually eat your meat?
   a) Well done
   b) Medium – rare

Institutional Review Board
American University of Beirut

16 FEB 2022

APPROVED

c) Rare
d) I don't eat meat

5. With the electricity cutoff and with the increase of food poisoning cases in Lebanon, did you shift from ordering medium- rare meat to order well done meat?
   a) Yes
   b) No

6. What food are you afraid the most to eat from outside your house (restaurants) during electricity cut-off?
   a) Burger/sandwiches
   b) Sushi
   c) Salads
   d) Everything
   e) Nothing

Section 4: Practices that could increase the risk of food poisoning

1. How often do you check the temperature of your fridge/freezer?
   a) Once/day
   b) Twice/day
   c) More than 3 times/day
   d) I don't check it

2. During electricity cut-off, do you take the temperature of food inside the fridge/freezer?
   a) Yes
   b) No

3. If electricity was off for more than 4 hours consecutive, what do you usually do with the refrigerated food?
   a) I take the temperature of the food and then decide to keep or not
   b) I smell and taste the food and then decide to keep or not
   c) I keep the fridge closed until the electricity is back again
   d) I discard all the food directly
   e) I don't do anything

Institutional Review Board
American University of Beirut

16 FEB 2022

APPROVED

4. After the economic crisis and electricity shortages, did you reduce your purchasing of certain food (chicken, meat, cheese, milk, fish)
    a) Yes
    b) No

5. If electricity was off for more than 4 hours consecutive, what do you usually do with the frozen food?
    a) Discard the food as a waste
    b) Consume/cook the food directly
    c) Transfer the food from fridge to freezer
    d) I take the temperature of the food and then decide to keep or not
    e) Keep the food inside the freezer and do nothing

6. When electricity is off for more than 4 hours. What is the best action to do regarding the following food items:

Food item	Fridge/Freezer	Discard	Keep
Leftover cooked meals	Fridge		
Raw meat/chicken	Fridge		
Pizza (any topping)	Fridge		
Soft cheeses (Halloumi, kashkavan, mozzarella)	Fridge		
Hard cheeses (cheddar, Swiss, parmesan)	Fridge		
Milk	Fridge		
Peanut butter	Fridge		
Opened mayonnaise-based dressing	Fridge		
Cooked rice, pasta, potato	Fridge		
Fresh vegetables (cut)	Fridge		
Fresh vegetables (uncut)	Fridge		
Bread	Fridge		
Egg products/custard	Fridge		
Thawed meat/ chicken with ice crystals	Freezer		
Thawed meat/ chicken without ice crystals	Freezer		

Institutional Review Board
American University of Beirut

16 FEB 2022

APPROVED

Thawed Ice cream	Freezer		

Institutional Review Board
American University of Beirut

16 FEB 2022

APPROVED
