# Supplementary material for: Beliefs, Practices, and Knowledge of Household Food Handlers Regarding the Impact of Electricity Outages on Food Safety: Findings from a National Cross-Sectional Study in Lebanon
Source: Foods. 2025 Mar 2;14(5):855. doi: 10.3390/foods14050855 (PMC11898753; doi:10.3390/foods14050855)
Supplement: Supplementary file 1 [file foods-14-00855-s001.zip › Supplementary Material File S4.pdf]

Appendix D: Two examples illustrating the assessment of the necessary assumptions for the chi-square test of independence

Example 1

Case Processing Summary

	Cases					
	Valid		Missing		Total	
	N	Percent	N	Percent	N	Percent
Are you the primary food handler in your household? * Beliefcategorical	571	100.0%	0	0.0%	571	100.0%

Are you the primary food handler in your household? * Beliefcategorical Crosstabulation

			Beliefcategorical		
			BadBelief	GoodBelief	Total
Are you the primary food handler in your household?	Yes	Count	210	52	262
		Expected Count	210.2	51.8	262.0
		% within Are you the primary food handler in your household?	80.2%	19.8%	100.0%
		% within Beliefcategorical	45.9%	46.0%	45.9%
		% of Total	36.8%	9.1%	45.9%
	No	Count	248	61	309
		Expected Count	247.8	61.2	309.0
		% within Are you the primary food handler in your household?	80.3%	19.7%	100.0%
		% within Beliefcategorical	54.1%	54.0%	54.1%
		% of Total	43.4%	10.7%	54.1%
Total		Count	458	113	571

	Expected Count	458.0	113.0	571.0
	% within Are you the primary food handler in your household?	80.2%	19.8%	100.0%
	% within Beliefcategorical	100.0%	100.0%	100.0%
	% of Total	80.2%	19.8%	100.0%

Chi-Square Tests

	Value	df	Asymptotic Significance (2-sided)	Exact Sig. (2-sided)	Exact Sig. (1-sided)
Pearson Chi-Square	.001[a]	1	.975		
Continuity Correction[b]	.000	1	1.000		
Likelihood Ratio	.001	1	.975		
Fisher's Exact Test				1.000	.529
Linear-by-Linear Association	.001	1	.975		
N of Valid Cases	571				

a. 0 cells (.0%) have expected count less than 5. The minimum expected count is 51.85.

b. Computed only for a 2x2 table

Example 2

Case Processing Summary

	Cases					
	Valid		Missing		Total	
	N	Percent	N	Percent	N	Percent
Gender * Knowledgecategorical	571	100.0%	0	0.0%	571	100.0%

Gender * Knowledgecategorical Crosstabulation

			Knowledgecategorical		
			BadKnowledge	GoodKnowledge	Total
Gender	female	Count	162	186	348
		Expected Count	155.4	192.6	348.0
		% within Gender	46.6%	53.4%	100.0%
		% within Knowledgecategorical	63.5%	58.9%	60.9%
		% of Total	28.4%	32.6%	60.9%
	male	Count	93	130	223
		Expected Count	99.6	123.4	223.0
		% within Gender	41.7%	58.3%	100.0%
		% within Knowledgecategorical	36.5%	41.1%	39.1%
		% of Total	16.3%	22.8%	39.1%
Total		Count	255	316	571
		Expected Count	255.0	316.0	571.0
		% within Gender	44.7%	55.3%	100.0%
		% within Knowledgecategorical	100.0%	100.0%	100.0%
		% of Total	44.7%	55.3%	100.0%

Chi-Square Tests

	Value	df	Asymptotic Significance (2-sided)	Exact Sig. (2-sided)	Exact Sig. (1-sided)
Pearson Chi-Square	1.292[a]	1	.256		
Continuity Correction[b]	1.104	1	.293		
Likelihood Ratio	1.295	1	.255		
Fisher's Exact Test				.263	.147
Linear-by-Linear Association	1.290	1	.256		
N of Valid Cases	571				

a. 0 cells (.0%) have expected count less than 5. The minimum expected count is 99.59.

b. Computed only for a 2x2 table
